# Rehabilitation Following Surgically Treated Distal Radius Fractures: Do Immobilization and Physiotherapy Affect the Outcome?

**DOI:** 10.7759/cureus.16230

**Published:** 2021-07-07

**Authors:** Kavyansh Bhan, Kamrul Hasan, Anjali S Pawar, Ronak Patel

**Affiliations:** 1 Trauma and Orthopaedics, Whipps Cross University Hospital, London, GBR; 2 Trauma and Orthopaedics, Barts Health NHS Trust, London, GBR; 3 Surgery, Whipps Cross University Hospital, London, GBR

**Keywords:** distal radius fractures, prolonged immobilization, physiotherapy intervention, hand therapy, orthopedic rehabilitation, wrist fractures

## Abstract

Distal radius fractures (DRF) are one of the most common fractures treated by orthopaedic surgeons around the globe. It has been estimated that the National Health Services (NHS) spends an average of £1375.34 per patient for surgical fixation of DRF with a volar locking plate as a day case. This figure climbs to £1983.39 if the same patient stays at the hospital overnight.^ ^Inpatient physiotherapy costs the NHS a staggering £82.03 per day, while each outpatient session with physiotherapy is £40.70 for the NHS. This means that a substantial amount is spent by the NHS on rehabilitation and physiotherapy for each DRF, whether fixed surgically or non-surgically. Post-operative rehabilitation involving initial immobilization followed by physiotherapy/hand therapy is an indispensable part of the total management concept of DRF. Most of the conservative management protocols also advocate a five-week immobilization followed by physiotherapy/hand therapy. Due to the fact that more than 50% of the patients with DRF are still employed, the impairment caused by a restriction of range of motion, duration of the sick leave and the effects of DRF on quality of life play a very important socio-economical role in the broadest sense. Patients are routinely referred to physiotherapists/hand therapists following DRF to improve the range of motion (ROM), manage pain, strengthen the wrist and develop full functionality to pre-injury levels. However, the real impact of supervised exercises and active physiotherapy in restoring mobility and strength to the fractured wrist is still not well understood. This article aims to review the existing literature and evidence base regarding the efficacy of immobilization and physiotherapy in improving the functional outcome of surgically treated DRFs.

## Introduction and background

Distal radius fractures (DRF) are one of the most common fractures treated by orthopaedic surgeons around the globe. It is the second most common fracture treated in the United States, with an estimated annual incidence of 643,000 [1]. In the United Kingdom (UK), DRF is considered to be the most common adult orthopaedic fracture [2]. It is the most common upper extremity fracture, accounting for one-fourth of all upper extremity fractures [3]. The incidence of DRF has only been rising across all age groups, with a documented rise in the incidence of 2.0% per annum in men and 3.4% per annum in women in the period 1999-2010. While the exact cause for this increase in incidence is unclear, multiple theories have attributed lifestyle influences (urban accounting for more as opposed to rural), rising life expectancy and higher incidence of osteoporosis [4]. A recent surge in engagement with sport-related activities may also be directly related to the rising incidence of DRF [3].

As the incidence of DRF rises, the short- and long-term costs also become apparent. It has been estimated that the National Health Services (NHS) spends an average of £1375.34 per patient for surgical fixation of DRF with a volar locking plate as a day case [5]. This figure climbs to £1983.39 if the same patient stays at the hospital overnight [5]. Inpatient physiotherapy costs the NHS a staggering £82.03 per day, while each outpatient session with physiotherapy is £40.70 for the NHS [5]. This means that a substantial amount is spent by the NHS on rehabilitation and physiotherapy for each DRF when fixed surgically.

Non-surgical treatment for DRF is largely dictated by Lafontaine criteria. In 1989, Lafontaine detailed five predictors for instability, namely age >60 years, dorsal angulation >20 degrees, dorsal comminution, fracture extension into the radiocarpal joint and associated ulnar fracture [6]. It was hypothesized that the presence of these predictors rendered a DRF unstable, hence necessitating surgical fixation [7]. The absence of these predictors, however, meant that the fracture could be treated conservatively, thereby requiring cast immobilization for five to six weeks. Multiple studies done since Lafontaine first described that the predictors have reinforced the importance of these predictors. However, recent studies have now stated that the only factors affecting the stability of DRF are dorsal comminution and age >60 years [8]. On the other hand, Song et al. in their meta-analysis have stated that there is no statistically significant difference in functional outcomes of operative vs nonoperative treatment for stable and unstable fractures alike [9].

According to AO (Arbeitsgemeinschaft für Osteosynthesefragen), the four principles of fracture fixation are fracture reduction, fracture fixation, preservation of blood supply and early mobilization. Interestingly, early mobilization is not routinely performed in DRF fixations. Although it is widely accepted that locking plate systems provide enough stability to unstable fractures to allow early mobilization, there is still no consensus on whether a ‘stable’ wrist should be mobilized after fixation, or if immobilized, how long should it be immobilized for [10]. Moreover, there are only a few published studies that investigate the benefits of shorter immobilization [10].

Post-operative rehabilitation involving initial immobilization followed by physiotherapy/hand therapy is an indispensable part of the total management concept following a DRF fixation. Most of the conservative management protocols also advocate a five-week immobilization followed by physiotherapy/hand therapy [10]. Due to the fact that more than 50% of the patients with DRF are still employed, the impairment caused by a restriction of range of motion (ROM), duration of the sick leave and the effects of DRF on quality of life play a very important socio-economical role in the broadest sense [11]. Patients are routinely referred to physiotherapists/hand therapists following DRF to improve the ROM, manage pain, strengthen the wrist and develop full functionality to pre-injury levels [12]. However, the real impact of exercises and active physiotherapy in restoring mobility and strength to the fractured wrist is still not well understood [12].

Given the current scenario of the COVID pandemic, it has become increasingly difficult for patients across the globe to seek medical advice in a timely manner. In such situations, the added burden of physiotherapy/hand therapy sessions and prolonged immobilizations may have a detrimental effect on the patient’s overall wellbeing. The main aim of this article is to critically analyze the available literature to determine the role immobilization and physiotherapy play in the outcome following surgically treated DRFs.

## Review

Although it is widely known that physiotherapy plays a positive role in helping regain the mobility of impaired extremities, its role in improving the functional outcome following operatively treated DRF is still not widely understood [12]. Moreover, whether a difference exists in patients undergoing supervised physiotherapy as opposed to home exercising programmes is even more unclear. Things are also complicated by the fact that there is no unanimous consensus on how long a wrist should be immobilized for operatively treated DRF [10].

Mobilization versus immobilization

It was back in 1814 that Colles had warned the doctors about the possible ill-effects of prolonged immobilization, including the possibility of the patient having an impaired wrist function [13]. Almost 200 years later, clinical studies on conservatively managed DRF have now suggested that shorter immobilization of three weeks instead of the widely practiced immobilization of five weeks leads to improved short-term outcomes and no increased risk of displacement of the fracture fragments [14]. A randomized control trial published in 2018 has suggested that immobilization periods of one week or three weeks produce far superior short-term functional outcomes when compared to patients who are immobilized for six weeks [15]. The same study has also suggested that there was no significant difference in the long-term outcomes of shorter immobilization versus long-term immobilization. This would suggest that short immobilization of one or three weeks improves functional outcomes in the short term and allows the patients to return to work earlier as opposed to longer immobilization periods of five or six weeks. Moreover, apart from functional comparisons, a prospective study by Kwan et al. has also suggested no significant difference in the radiological parameters of articular congruency in operatively managed DRF with immediate post-op mobilizations [16]. Another case series by Osada et al. which involved 49 patients treated surgically with volar locking plates and immediate post-op mobilization without any routine physiotherapy showed 98% of the patients had ‘excellent’ outcomes according to the modified Green O’Brien score with a mean DASH of six points at a 12 month follow up [17]. This also suggests that routine recommendation of physiotherapy after a DRF fixation may not be ideally needed. A larger study of 72 patients by Duprat et al. compared two-week splint immobilization to the immediate post-op mobilization of surgically fixed DRF and noted no statistically significant difference in ROM and grip strength of the patients of these two groups at a three month follow up period [18]. Another point of note is that Duprat et al. also did not notice any complications like displacement or loss of reduction in their study of these 72 patients. A randomized control trial (RCT) by Lozano-Calderon et al. compared surgically treated DRF managed with immobilization for six weeks against surgically treated DRF which were encouraged to mobilize within two weeks post-operatively [19]. There was also no supervised physiotherapy or hand therapy, and patients only had a single session in which specific wrist exercises were shown to them. A follow-up at three months and six months demonstrated no significant difference amongst the outcomes of these two groups with regards to the ROM, grip strength, pain or the radiological parameters. A recent study by Andrade-Silva et al. evaluated the pain outcomes on a visual analogue scale (VAS) for DRF patients treated surgically with immediate post-op mobilization and DRF patients treated surgically followed by a two-week immobilization in removable splints [20]. Neither of the group received any kind of supervised physiotherapy. Interestingly, six months follow-ups suggested no statistically significant difference in the VAS scores of the two groups. Moreover, patients who were allowed to mobilize immediately post-operatively did not exhibit a higher demand for painkillers compared to those who had initial two-week immobilization. One of the prime concerns of allowing early mobilization is a possible need for higher doses of opioids to keep pain under control for patients allowed to mobilize early post-surgery. However, the study by Andrade-Silva et al. has suggested that the pain scores are similar irrespective of the period of immobilization [20].

Although most of the studies discussed above do have their shortcomings in the form of different study designs, different rehabilitation protocols or varied sample size calculations, the gist of these studies is that prolonged immobilization following operatively treated DRFs may not be entirely necessary. Moreover, the popular belief that early mobilization is associated with increased pain may also be unfounded.

Physiotherapy versus no physiotherapy

Patients with DRFs across the globe are routinely referred to hand therapy/physiotherapy after a varying period of immobilization. However, whether supervised physiotherapy or active wrist exercises have a positive impact on regaining the wrist function and ROM when compared to sole home exercising programmes is still unclear [11]. Souer et al. in an RCT of almost 100 patients concluded that supervised physiotherapy resulted in inferior functional outcomes when compared to independent home exercise regimens [21]. They suggested that one of the possible reasons for superior outcome of home exercise regimen may be due to the fact that therapists are overly cautious which may limit the patients in performing exercises beyond the point at which it becomes painful. This may lead to a slower or delayed recovery and thus the inferior outcome of supervised therapies. Krischak et al. conducted a randomized controlled cohort study of 48 patients divided into two groups. The first group underwent an unassisted home exercise programme while the second group had 12 supervised hand therapy sessions under the care of a hand therapist. At the six-week mark, they concluded that the patients who had unsupervised and unassisted home exercise programme had a significantly better function of the wrist as opposed to the patients who were under the care of hand therapists [22]. The unsupervised patients had almost 50% better functional outcomes when comparing the grip strength and the ROM in flexion and extension. Another study by Kay et al. of 56 patients of DRF treated with a cast or K-wire fixation, divided the patients into an experimental group of the physiotherapist-directed rehabilitation programme and a control group of nil intervention following the immobilization [23]. Interestingly, no statistically significant difference was found in either of the groups with regards to the wrist extension at a three-week mark or the grip strength and other ROMs at the six-week mark. This may suggest that a physiotherapist-directed programme of rehabilitation may have no benefit in actually improving the ROM and grip strength of the patients undergoing treatment for DRFs. Bruder et al. reported the outcomes of their multi-centre RCT involving 33 patients of DRF managed conservatively in a cast. Their trial has shown no evident benefit at 24 weeks in the ROM of patients receiving a progressive exercise programme under physiotherapists as opposed to structured advice alone by the physiotherapists for DRF patients treated after six weeks of immobilization [12]. A prospective randomized control trial by Valdes et al. evaluated the efficacy of therapist-supervised hand therapy versus home therapy for 50 patients who underwent Volar locking Plate fixation for DRF in a multi-centre study [24]. They concluded that there were no differences in mean Patient-Rated Hand Wrist Evaluation (PRHWE) scores of both groups at follow-up of six months. They also did not note any statistically significant difference in the wrist ROM between the comparing groups at six months follow-up. They, however, suggested that the use of supervised hand therapy be reserved for patients with subpar or suboptimal fixation or patients with complications after a volar locking plate fixation including but not limited to complex regional pain syndrome or carpal tunnel syndrome [24].

The evidence discussed above suggests that most patients may achieve satisfactory results whether they attend a supervised physiotherapy programme or a home exercise programme. This is also supported by the Wrist and Radius Injury Surgical Trial published in 2020, which suggests that most patients will have an improvement in their ROM irrespective of whether they attend supervised hand therapy programmes or follow simple home exercises post immobilization [25]. Most of the published literature does advise some form of exercise programme, either supervised or unsupervised to improve the ROM and grip strength following DRF. However, for many patients, simply performing the activities of daily living may well be enough activity that is needed to achieve the rehabilitation goals of ROM and grip strength amongst others [26]. Moreover, in the current scenario of COVID 19, it is now widely recommended to treat DRF as a non-urgent fracture in a conservative or non-operative manner. The threshold for accepting malunion, stiffness and reduced ROM has increased manifold, and the need for physiotherapy in such situations is thus questionable [27]. Also, most of the studies discussed in this review have suggested that a home exercise programme is as effective as supervised physiotherapy/hand therapy and provides similar functional outcomes [28]. Multiple studies have suggested a period of three-week immobilization as opposed to the more routinely practiced five- or six-week immobilization for the sole reason of pain relief rather than prevention of displacement of the fracture fragments [29,30].

**Table 1:** Summary of Randomized Controlled Trials Reviewed RCT: randomized controlled trial.

Study	N	Study design	Immobilization	Rehabilitation
Bruder et al. [12]	33	RCT	Experiment (n=19) and control (n=14): plaster cast for 6 weeks	6 weeks after injury
Watson et al. [15]	133	RCT	1 week (n=46): palmar splint 3 weeks (n=41): forearm cast 6 weeks (n=46): forearm cast	1 week after surgery or 3 weeks after surgery or 6 weeks after surgery
Lozano-Calderón et al. [19]	60	RCT	Early (n=30) and late (n=30) mobilization: removable thermoplastic palmar splint for 6 weeks	Early mobilization: immediate post-op exercises (splint was taken off only for exercises) late motion: 6 weeks after surgery
Andrade-Silva et al. [20]	39	RCT	Group I (No splint): NIL group II (splint group): Palmar plaster splint for 2 weeks	Group I: Immediate post-op exercises group II: 2 weeks after surgery
Souer et al. [21]	94	RCT	Experiment (n=46): NIL immobilization control (n=48): wrist splint until free finger and forearm motion	Experiment: Immediate post-op exercises control: after free finger and forearm motion
Krischak et al. [22]	48	RCT	Experiment (n=23) and control (n=23): forearm splint which was removed for therapy after 1 week	Experiment and control: 1 week after surgery
Valdes et al. [24]	50	RCT	Experiment (n=22) and control (n=28): NIL immobilization	The study compared home therapy vs supervised therapy

## Conclusions

Distal radius fracture is undoubtedly one of the most common fractures treated by an orthopaedic surgeon. Controversies have long spanned the breadth of treatment options and rehabilitation protocols available for DRFs. In this time of the pandemic, when it is being widely recommended to treat all kinds of DRFs irrespective of the fracture geometry or age of the patient in a conservative or non-operative manner, the feasibility and need for physiotherapy sessions after operative treatments of DRF become questionable. Patients are being widely recommended self-removal of plasters or splints and follow-up through the means of telemedicine to keep the number of visits to healthcare facilities to a bare minimum. In such times, one really needs to ask whether one should recommend patients to attend physiotherapy sessions for non-life-threatening fractures like DRF. The effectiveness of physiotherapy, whether supervised or unsupervised, is still unclear. Multiple randomized trials have compared the functional outcomes of patients having physiotherapy versus home exercise programmes. Although most of the studies are level II evidence, they have suggested that a home exercise programme is as effective as supervised physiotherapy/hand therapy and provides similar functional outcomes. However, further RCTs are essential to evaluate the exact role of physiotherapy and the need for physiotherapy in the rehabilitation of DRFs.

Although patients are routinely immobilized after DRFs, whether treated surgically or non-surgically, the exact duration of cast immobilization remains disputed. Even though multiple authors have suggested a short immobilization of three weeks post-fixation, many have recommended immediate post-op mobilization, thereby improving the short-term outcome with no difference in the long-term outcome. Also, multiple studies have suggested a period of three-week immobilization as opposed to the more routinely practiced five- or six-week immobilization for the sole reason of pain relief rather than prevention of displacement of the fracture fragments. The period of immobilization is important since it also directly affects the possible date of return to work for patients with DRF. Moreover, COVID 19 pandemic has created unprecedented challenges for the healthcare system, and an added burden of prolonged immobilization with the need for follow-up visits to healthcare facilities for the sole reason of the removal of plaster/cast would definitely make the referring surgeon rethink the need and merits of immobilization. To date, the literature is not suggestive of any difference in functional outcome after three months post-surgery between the varying periods of immobilization. Hence, the treating surgeon needs to adapt to the ever-changing global scenario and advise physiotherapy and immobilization according to the patient's needs, access to healthcare, the overall safety of the patient and compliance to the post-surgical restrictions.
